# FGF-Receptors and PD-L1 in Anaplastic and Poorly Differentiated Thyroid Cancer: Evaluation of the Preclinical Rationale

**DOI:** 10.3389/fendo.2021.712107

**Published:** 2021-08-12

**Authors:** Pia Adam, Stefan Kircher, Iuliu Sbiera, Viktoria Florentine Koehler, Elke Berg, Thomas Knösel, Benjamin Sandner, Wiebke Kristin Fenske, Hendrik Bläker, Constantin Smaxwil, Andreas Zielke, Bence Sipos, Stephanie Allelein, Matthias Schott, Christine Dierks, Christine Spitzweg, Martin Fassnacht, Matthias Kroiss

**Affiliations:** ^1^Department of Internal Medicine I, Division of Endocrinology/Diabetology, University of Würzburg, Würzburg, Germany; ^2^Institute of Pathology Würzburg, University of Würzburg, Würzburg, Germany; ^3^Comprehensive Cancer Center Mainfranken, University of Würzburg, Würzburg, Germany; ^4^Department of Internal Medicine IV, University Hospital of Munich, LMU Munich, Munich, Germany; ^5^Department of Medicine I, Goethe University Hospital, Frankfurt, Germany; ^6^Institute of Pathology LMU, University Hospital of Munich, LMU Munich, Munich, Germany; ^7^Department of Internal Medicine III, University Hospital of Leipzig, Leipzig, Germany; ^8^Department of Internal Medicine I, Division of Endocrinology/Diabetology, University of Bonn, Bonn, Germany; ^9^Institute of Pathology Leipzig, University Hospital of Leipzig, Leipzig, Germany; ^10^Department of Endocrine Surgery, Diakonie-Klinikum Stuttgart, Stuttgart, Germany; ^11^Medical Oncology and Pulmonology, University Hospital, Tübingen, Germany; ^12^Division for Specific Endocrinology, Medical Faculty, University of Düsseldorf, Düsseldorf, Germany; ^13^Department of Internal Medicine IV, Division of Oncology and Hematology, University of Halle (Saale), Halle (Saale), Germany

**Keywords:** tyrosine kinase inhibitor (TKI), immune checkpoint inhibitor (ICI), immunohistochemistry, immunotherapy, PD-L1, FGFR

## Abstract

**Background:**

Treatment options for poorly differentiated (PDTC) and anaplastic (ATC) thyroid carcinoma are unsatisfactory and prognosis is generally poor. Lenvatinib (LEN), a multi-tyrosine kinase inhibitor targeting fibroblast growth factor receptors (FGFR) 1-4 is approved for advanced radioiodine refractory thyroid carcinoma, but response to single agent is poor in ATC. Recent reports of combining LEN with PD-1 inhibitor pembrolizumab (PEM) are promising.

**Materials and Methods:**

Primary ATC (n=93) and PDTC (n=47) tissue samples diagnosed 1997-2019 at five German tertiary care centers were assessed for PD-L1 expression by immunohistochemistry using Tumor Proportion Score (TPS). FGFR 1-4 mRNA was quantified in 31 ATC and 14 PDTC with RNAscope *in-situ* hybridization. Normal thyroid tissue (NT) and papillary thyroid carcinoma (PTC) served as controls. Disease specific survival (DSS) was the primary outcome variable.

**Results:**

PD-L1 TPS≥50% was observed in 42% of ATC and 26% of PDTC specimens. Mean PD-L1 expression was significantly higher in ATC (TPS 30%) than in PDTC (5%; p<0.01) and NT (0%, p<0.001). 53% of PDTC samples had PD-L1 expression ≤5%. FGFR mRNA expression was generally low in all samples but combined FGFR1-4 expression was significantly higher in PDTC and ATC compared to NT (each p<0.001). No impact of PD-L1 and FGFR 1-4 expression was observed on DSS.

**Conclusion:**

High tumoral expression of PD-L1 in a large proportion of ATCs and a subgroup of PDTCs provides a rationale for immune checkpoint inhibition. FGFR expression is low thyroid tumor cells. The clinically observed synergism of PEM with LEN may be caused by immune modulation.

## Introduction

Anaplastic (ATC) and poorly differentiated thyroid carcinoma (PDTC) are orphan diseases which account for 1-2% and 2-15% % among all thyroid malignancies (1, 2). While treatment of DTC is well established and 5-year survival rates are above 90% (3), the management of PDTC and ATC is unsatisfactory and prognosis generally poor with a median overall survival of only six months for ATC patients (4, 5). Current guidelines recommend surgery in ATC cases (stage IVA and IVB) and a careful evaluation of surgical options in stage IVC cases (6). Surgery can be followed by additive chemoradiation therapy to improve locoregional control and overall outcome (7, 8). Nonsurgical treatment options include chemotherapy, palliative radiotherapy, systemic therapy or best supportive care (6). The combination of BRAF inhibitor dabrafenib and MEK inhibitor trametinib for BRAF V600E-mutated ATC poses a recent breakthrough with an overall response rate of 69% (9, 10). For stage IVC non-BRAF V600E-mutated cases guidelines recommend evaluation of PD-L1 status and treatment with checkpoint inhibitors as an alternative to chemotherapy and/or radiation (6). The prognosis of PDTC is more favorable with a 5-year survival rate of 66% because some PDTC are accessible to radioiodine treatment (11, 12). Secondary resistance to radioiodine therapy limit a curative approach in advanced cases (13). Lenvatinib (LEN) is a multi-tyrosine kinase inhibitor (TKI) of VEGFR1-3, FGFR1-4, PDGFR-α, RET and c-kit and approved for the treatment of progressive radioiodine refractory DTC and radioiodine refractory PDTC (14). Nevertheless, important practical issues in the use of tyrosine kinase inhibitors are still unsolved (15, 16).

In an observational study Iwasaki et al. compared treatment with LEN (n=16) and palliative therapy (n=16) in 32 stage IVC ATC patients. The median overall survival (OS) time of patients treated with LEN was 4.2 months while patients receiving palliative therapy had a median overall survival of only 2.0 months (17). In a very recent study of post-marketing registry data, LEN showed an objective response in 44% of ATC patients which was, however, short-lived with a median overall survival of 101 days (18).

Although many of the TKIs currently used in the treatment of thyroid carcinomas refractory to radioiodine share common, e.g., antiangiogenetic TKI activity it has been speculated that the superior clinical response of LEN in these rare thyroid carcinomas may be attributable to its ability to also target FGFR 1-4 (19).

A study that used immunohistochemistry to investigate FGFR4 expression in 12 ATC patients suggested that FGFR4 expression may predict response to LEN (20).

The immune checkpoint inhibitor (ICI) pembrolizumab (PEM) is a programmed death 1 (PD-1) inhibitor approved for numerous types of cancer such as non-small cell lung carcinoma (NSCLC), renal cell carcinoma (RCC), hepatocellular carcinoma (HCC) and head and neck squamous cell cancer (HNSCC) (21–24). Tumoral tissue expression of its ligand PD-L1 as assessed by immunohistochemistry has been proposed as a biomarker and for some tumor entities disease-specific cut-offs have been suggested (25). As part of a phase I/II study 42 ATC patients were treated with PD-1 inhibitor spartalizumab and showed higher response rates in PD-L1-positive versus PD-L1 negative ATC patients with highest rate of response in patients with PD-L1 ≥ 50% (26). In ATC, a phase II trial of single agent PEM or a combination with chemoradiotherapy was prematurely terminated due to rapid fatal outcome in the three patients investigated (27). Trials in multiple disease entities are ongoing including a trial in patients with radioiodine refractory thyroid carcinoma without prior TKI treatment from which preliminary positive results have been reported (NCT02973997).

Addition of PEM after prior failure to TKI therapy with LEN, dabrafenib alone or in combination with trametinib has been reported in a retrospective single center study. Partial response (PR) was seen in 43% but with a very short median PFS of 3 months and a median overall survival (OS) of 7 months only (8). In a recent series of metastatic ATC and PDTC negative for the BRAF V600E mutation, 8 patients received a combination therapy of LEN and PEM for a maximum of 40 months after failing chemotherapy, radiation or radioiodine therapy. The combination treatment was not only well tolerated with 50% (3/6) of ATC patients still on therapy at data cutoff but also led to complete response (CR) in 66% (4/6) of ATC patients after 7, 10 and 12 months and PR in 75% of patients after three to four months of treatment (28). Range of PD-L1 expression was 1%-90% and patients with a PD-L1 expression greater than 50% (5/8) responded best to combination therapy (28). There was no association of PD-1 expression or frequency of tumor infiltrating lymphocytes (TILs) with treatment response (28).

The primary aim of this study is to evaluate a potential molecular rationale for a treatment of ATC and PDTC patients with lenvatinib, pembrolizumab or a combination of both. Secondary aims were the investigation of FGFR and PD-L1 expression as prognostic markers and potential marker of treatment response. As an exploratory aim, we describe the clinical benefit of patients treated with these substances as a monotherapy or in a combined regimen.

## Materials and Methods

### Setting

This study was conducted as part of the German Study Group for rare malignant tumors of the thyroid and parathyroid glands. The study was approved by the ethics committee of the University of Würzburg (96/13) and subsequently by the ethics committees of all participating centers. All patients provided written informed consent. Prospectively and retrospectively collected data and formalin-fixed paraffin embedded (FFPE) tissue samples of patients diagnosed with ATC and PDTC between 1997 and 2019 were obtained from five German tertiary care centers.

### Samples and Data Acquisition

Adult patients with local diagnosis of ATC or PDTC at histopathologic examination of sections or biopsy of the primary tumor were eligible. Archival anonymized papillary thyroid carcinoma (PTC) and normal thyroid (NT) tissue from the institute of pathology, University of Würzburg, served as control.

Clinical data such as the date of diagnosis, tumor stage at initial diagnosis, treatments including surgical interventions, radioiodine treatment and systemic therapies (e.g., cytotoxic chemotherapy, radiotherapy, TKI- and/or ICI-therapy), metastatic sites and number of treatment lines, were recorded by trained personnel at all sites. Tumor stage was recorded according to UICC classification (TNM Classification of Malignant Tumours, 8^th^ edition, 2017) and determined for PDTC and ATC respectively.

Study endpoints were disease-related death or time interval from diagnosis to last follow-up alive. Treatment and follow-up of patients was done according to standard of care at participating centers.

### Histopathological Review

The diagnosis of ATC and PDTC in archival FFPE tissue samples (n=140) was verified by a single endocrine pathologist (SK). According to the current WHO Classification of Tumours of Endocrine organs (4^th^ edition, volume 10, 2017).

### Immunohistochemistry

All specimens were processed by immunohistochemistry and RNAscope^®^ *in situ* hybridization within two weeks after sectioning. Full FFPE sections of primary tumor tissue (n=93 ATC; n=47 PDTC) mounted on slides were deparaffinized, rehydrated and antigen retrieval was performed in target retrieval solution (Target Retrieval Solution, Citrate pH 6.1 [10x], Dako, CA, USA; 1:10 dilution) under pressure for 4 minutes. Following deparaffinization, steps were carried out with a Freedom EVO 200 base unit (TECAN Trading AG, Switzerland). Tissue sections were incubated with primary antibody (PD-L1 [28-8] rabbit monoclonal antibody 438R-15-ASR; Cell Marque, CA, USA; 1:100 dilution; Antibody Diluent, Dako REAL) for 1h at RT. Signal amplification was achieved by the HRP Kit (HPR HiDef 2-Step Polymer Detection Kit, medac GmbH, Germany) for 40 min and developed for 10 min with Histofine (Histofine DAB-2V, Nichirei Biosciences Inc., Japan). Nuclei were counterstained using Mayer’s hematoxylin for 3 min and blued for 10 min in running tap water. Following dehydration, slides were mounted by a Tissue-Tek (Sakura Finetek, Inc., CA, USA).

### RNAscope *In Situ* Hybridization

Given the limited reliability of immunohistochemistry for FGFRs due to their high degree of homology, RNAscope was used to study expression of FGFR 1-4. Tumor samples (ATC: n=31; PDTC: n=14) were cut to 2-μm thickness, deparaffinized in xylene, and rehydrated in a graded alcohol series. Fixation, permeabilization and protease digestion were achieved by treatment with hydrogen peroxide (322335, Advanced Cell Diagnostics [ACDbio], CA, USA) at RT for 10 min, target retrieval reagent (322000, ACDbio) under pressure for 15 min and protease plus (322331, ACDbio) at 40°C for 20 min. FGFR 1-4 probes (Hs-FGFR1, 310071; Hs-FGFR2, 311171; Hs-FGFR3, 310791; Hs-FGFR4-CD5, 412301, ACDbio) were then hybridized at 40°C for 2h. Slides were treated with Amplifier 1 (322311, ACDbio) and Amplifier 3 (22313, ACDbio) at 40°C for 45 min, with Amplifier 2 (322312, ACDbio) and Amplifier 4 (322314, ACDbio) at 40°C for 20 min, and with Amplifier 5 (322315, ACDbio) for 1h and Amplifier 6 (322316, ACDbio) for 20 min at RT. In between the amplification steps, slides were washed in wash buffer (310091, ACDbio) for 4 min, each. Equal volumes of DAB-A (DAB-A, 320052, ACDbio) and DAB-B (DAB-B, 320053, ACDbio) were mixed and pipetted on the slides. After incubation at RT for 10 minutes slides were put in hematoxylin, dehydrated and mounted. 

### Semi-Quantitative Analysis of PD-L1 and FGFR 1-4 Expression

PD-L1 slides were visually scored using an AxioScope.A1 microscope (Carl Zeiss AG, Germany). PD-L1 expression was evaluated using a semi-quantitative scoring system based on the proportion of stained tumor cells according to Tumor Proportion Score (TPS).

FGFR1-4 images were assessed with Aperio VERSA microscope (Leica Biosystems, Germany). Manual counting of cell nuclei and RNA was performed with ImageJ-win64 (Fiji, GitHub enterprise, CA, USA), and quantified as the number of FGFR 1-4 mRNA per cell.

### Statistical Analysis

DSS from diagnosis was estimated using the Kaplan-Meier method and groups were compared by the log-rank test. For data with non-normal distribution we used Mann-Whitney *U* test. Kruskal-Wallis test was used for comparison among groups for non-nominal distributed variables. Assessment of risk factors was performed by using the Cox proportional hazard regression model (forward and backward step-up regression). P values <0.05 were considered statistically significant. Statistical analyses were performed with SPSS Version 26 (IBM, Chicago, IL, USA). GraphPad Prism 7.0 (GraphPad Software, San Diego, CA, USA) and Microsoft Office Excel 2010 were used for graphical presentation and additional analyses.

## Results

### Clinical Characteristics

93 patients (40 male, 53 female) with histological evidence of ATC and 47 patients (17 male, 30 female) with poorly differentiated thyroid carcinoma treated at five German tertiary care centers were included. Baseline clinical characteristics of the study population are shown in **Table 1**. In brief, median age at primary diagnosis of ATC patients was 69 years (range 29-95) and 63 years (range 16-86) for patients with PDTC. At the time of initial diagnosis 47 patients with ATC (51%) and 13 patients with PDTC (28%) had local regional lymph node metastases. Disease was restricted to the thyroid gland (stage IVA) at diagnosis in one single ATC patient whereas 52% had distant metastasis (stage IVC). 15 PDTC patients had distant metastases at first diagnosis, four of whom were <55 years (UICC stage II) and 11 aged ≥55 years (UICC stage IVB).

**Table 1:** Patient characteristics of the study cohort.

Patient characteristics	No. of patients ATC (%)	No. of patients PDTC (%)
Number of patients	93	47
Male sex	40 (43)	17 (36)
Median age at diagnosis (range), in years	69 (29-95)	63 (16-86)
Median size of primary tumor (range), in mm	55 (8-105)	46.5 (12-95)
Not reported	18 (19)	5 (11)
Initial tumor stage		
T		
pT1	1 (1)	2 (4)
pT2	1 (1)	10 (21)
pT3	11 (12)	24 (51)
pT4	78 (84)	10 (21)
pTx	2 (2)	1 (2)
N		
pN0	19 (20)	19 (40)
pN1	47 (51)	13 (28)
pNx	27 (29)	15 (32)
M		
cM0	32 (34)	26 (55)
cM1	48 (52)	15 (32)
cMx	13 (14)	6 (13)
UICC		
I	–	13 (28)
II	–	17 (36)
III	–	3 (6)
IVA	1 (1)	2 (4)
IVB	41 (44)	11 (23)
IVC	48 (52)	–
Not available	3 (3)	1 (2)
Sites of metastases at baseline		
local regional lymph nodes	47 (51)	13 (28)
mediastinal lymph nodes	11 (12)	0 (0)
lung	41 (44)	13 (28)
liver	5 (5)	0 (0)
bone	9 (10)	3 (6)
pleura	2 (2)	3 (6)
heart	1 (1)	0 (0)
adrenal gland	1 (1)	0 (0)
thymus	0 (0)	1 (2)
brain	0 (0)	0 (0)

### Expression of PD-L1 in ATC and PDTC

**Figure 1:**
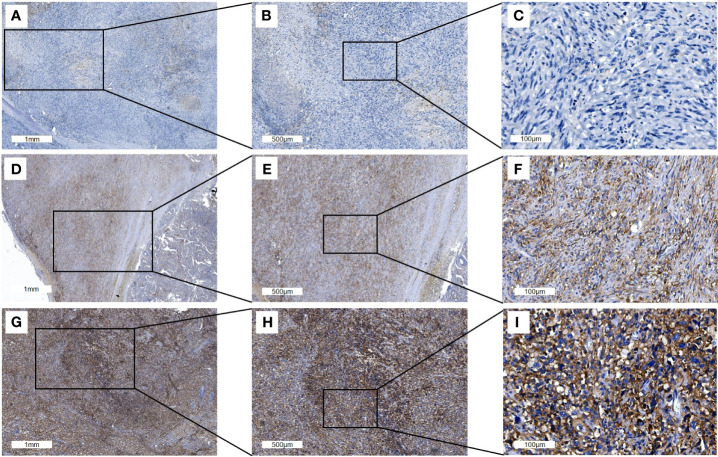
Representative PD-L1 immunohistochemistry staining of full ATC FFPE sections. Three different tissue samples stained with PD-L1 antibody are shown with an overview of the tissue sample (**A, D, G**; scale bars: 1mm), at 2x magnification (**B, E, H**; scale bars: 500μm) and 10x magnification (**C, F, I**; scale bars: 100μm). The PD-L1 TPS in **(A–C)** is 1% and 50% in D-F while 95% in **(G–I)**.

**Figure 2:**
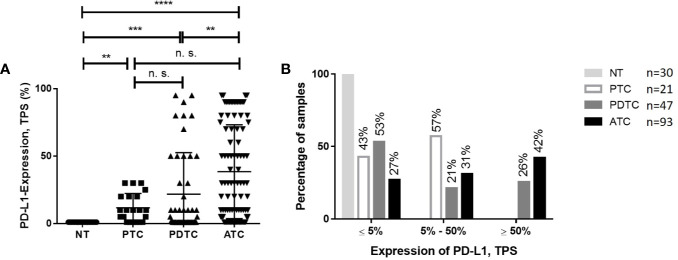
Comparison of PD-L1 expression in ATC or PDTC and PTC or NT. **(A)** PD-L1 expression as assessed by TPS was significantly higher in ATC compared to PDTC and tumor specimens compared to normal thyroid (NT). No significant differences (n. s. = not significant) could be observed in ATC and PTC and PDTC and PTC. **(B)** Proportion of PD-L1 TPS categories in PDTC and ATC. 42% of ATC and 26% of PDTC specimen show PD-L1 TPS ≥50%. **p < 0.01, ***p < 0.001, ****p < 0.0001.

The proportion of PD-L1 expression in tumor cells differed widely both in ATC and PDTC samples (**Figure 1**) and was heterogeneous within samples which is accounted for in the semiquantitative TPS. Median PD-L1 TPS was 30% (range 0-95) in ATC (n=93) compared to 5% (range 0-95) in PDTC (n=47, p<0.01, **Figure 2**) and was significantly higher in ATC (p<0.0001) and PDTC (p<0.001) compared to normal thyroid tissue samples (n=30). 39 (42%) of ATC samples and 12 (26%) of PDTC specimens expressed PD-L1 in ≥50% of tumor cells, while none of the PTC samples showed PD-L1 TPS of ≥50%. 27% of ATC samples and 53% of PDTC samples had PD-L1 TPS ≤5%. PD-L1 expression could not be detected in normal thyroid tissue (n=30). Differentiated PTC specimens (n=21) showed median PD-L1 expression of 10% (range 0-30).

### Expression of FGFR 1-4 in ATC and PDTC

**Figure 3:**
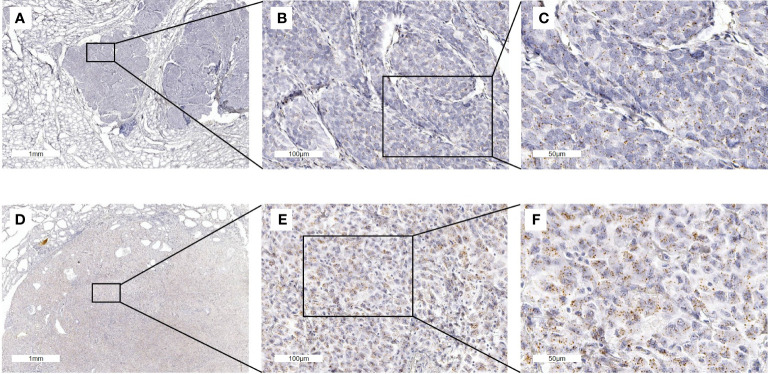
FGFR1 mRNA *in situ* hybridization staining of full ATC and PDTC FFPE sections. **(A–C)** FGFR1 RNAscope *in situ* hybridization in a PDTC tissue sample. FGFR1 expression is 1.9 mRNA/nucleus. **(D–F)** FGFR1 expression in an ATC tissue sample (5.4 mRNA/nucleus).

**Figure 4:**
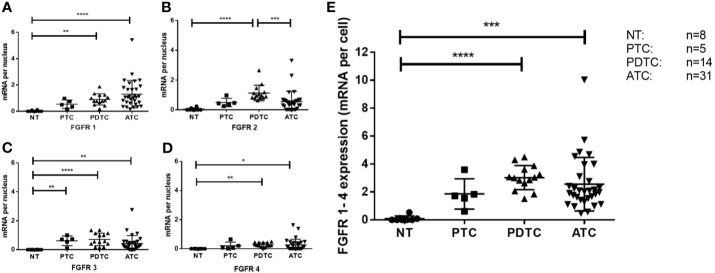
FGFR expression in ATC, PDTC, PTC and NT. FGFR1 **(A)**, FGFR2 **(B)**, FGFR3 **(C)** and FGFR4 **(D)** was detected at low levels in all samples studied. Significant differences are indicated. Combined scoring of FGFR1-4 expression in ATC and PDTC **(E)** is significantly higher compared to normal thyroid (NT) tissue. *p < 0.1, **p < 0.01, ***p < 0.001, ****p < 0.0001.

Expression of FGFR mRNA was generally low in all investigated samples (**Figure 3**). Median expression of FGFR 1 was 1.06 (range 0.16-5.42) mRNA/cell for ATC (n=31) tissue, 0.81 (range 0.18-1.87) mRNA/cell for PDTC (n=14) specimens, and 0.5 (range 0.15-0.97) mRNA/cell for PTC (n=5) tissue (**Figure 4**). Data on FGFR 2 and 3 are shown in **Table 2**. FGFR 4 exhibited the lowest median expression in all types of thyroid carcinoma (ATC: 0.14 mRNA/cell; PDTC: 0.24 mRNA/cell; PTC: 0.08 mRNA/cell). In comparison to normal thyroid tissue, panFGFR expression (sum of FGFR1–4) was significantly higher in ATC (p<0.001) and PDTC (p<0.0001) tissue (**Figure 4**). Median panFGFR expression was 2 mRNAs/cell in ATC and 3 mRNAs/cell in PDTC tissue. Normal thyroid tissue (n=8) expressed FGFR 1-4 at lowest levels with median expression of 0.0 mRNA/cell, respectively. FGFR 1-4 expression was not significantly different between ATC/PDTC and PTC tissue specimens.

**Table 2:** Median expression of FGFR 1-4 in ATC, PDTC and PTC.

FGFR expression (mRNA/cell)	ATC	PDTC	PTC	*P*
	(n=31)	(n=14)	(n=8)	(Kruskal-Wallis)
FGFR 1 (range)	1.06 (0.16-5.32)	0.81 (0.18-1.87)	0.5 (0.15-0.97)	0.079
*P* (Kruskal-Wallis*)	*P*=1.0			
FGFR 2 (range)	0.38 (0.0-3.3)	0.96 (0.64-2.65)	0.43 (0.25-0.97)	**<0.0001**
*P* (Kruskal-Wallis*)	***P*=0.001**			
FGFR 3 (range)	0.31 (0.0-2.75)	0.58 (0.14-1.36)	0.71 (0.09-1.0)	**0.042**
*P* (Kruskal-Wallis*)	*P*=0.168			
FGFR 4 (range)	0.14 (0.0-1.62)	0.24 (0.06-0.49)	0.08 (0.02-0.65)	0.169
*P* (Kruskal-Wallis*)	*P*=0.296			
panFGFR (range)	2.03 (0.47-10.03)	3.02 (1.52-4.5)	1.72 (0.61-3.6)	0.058
*P* (Kruskal-Wallis*)	*P*=0.532			

*p-values of Bonferroni testing for pairwise comparisons between ATC and PDTC are indicated.

### Disease-Specific Survival and Tumor-Specific Therapy

83 patients (89%) died due to ATC, 3 (3%) patients with ATC deceased due to causes other than ATC and 7 (8%) patients were still alive at last follow-up. 14 PDTC patients (30%) died from advanced PDTC. In 2 PDTC patients the cause of death was unknown and 31 (66%) were still alive at last follow-up. In 4 (4%) ATC and 4 (9%) PDTC patients, there was no evidence of disease at last follow-up indicating complete remission. Median overall survival was 6.4 months for patients diagnosed with ATC and 29.1 months for patients diagnosed with PDTC. Disease-specific survival was 5.4 (ATC) and 24.0 months (PDTC) in a median follow-up of 6 (ATC) and 28 (PDTC) months.

### Therapy With TKI and/or ICI

Tumor-specific therapy was consistent with previous publications on ATC (4) and is summarized in **Table 3**. Targeted therapies with tyrosine kinase inhibitors (TKI) and/or immune checkpoint inhibitors (ICI) were administered in 15 (16%) of ATC and 13 (28%) of PDTC patients. Patients were treated with LEN (n=3 ATC, n=9 PDTC), PEM (n=2 ATC, n=1 PDTC), LEN + PEM (n=1 ATC, n=2 PDTC) and other TKIs (n=10 ATC; n=8 PDTC). Two PDTC patients received three different lines of targeted therapy, another two PDTC patients were treated with two different TKIs (LEN followed by sorafenib and LEN followed by cabozantinib), and one ATC and one PDTC patient received two lines of targeted therapy.

**Table 3:** Therapeutic regimens in patients with ATC or PDTC.

Therapeutic regimen	No. of patients ATC (%)	No. of patients PDTC (%)
Primary surgery		
One-stage thyroidectomy	39 (42)	21 (45)
Two-stage thyroidectomy	7 (8)	12 (26)
Hemithyroidectomy	21 (23)	9 (19)
debulking surgery	13 (14)	0 (0)
biopsy	8 (9)	0 (0)
only explorative surgery	5 (5)	3 (6)
Not reported	0 (0)	2 (4)
Resection status		
R0	11 (12)	25 (53)
R1	37 (40)	10 (21)
R2	34 (37)	2 (4)
Rx	11 (12)	10 (21)
Radioiodine treatment (RIT)	6 (7)	36 (77)
Median number of RIT (range)	1 (1-1)	1 (1-11)
Median cum. Dose (GBq) (range)	3.36 (2.7-7.4)	6.65 (1.2-74.2)
Dose not available	1 (1)	0 (0)
Radiochemotherapy (RCT)	38 (42)	4 (9)
not available	2 (2)	0 (0)
External beam radiation		
Neck region	79 (85)	16 (34)
Local palliative	52 (56)	7 (15)
Median cum. Dose (Gy) (range)	55.0 (4-105.6)	60.5 (50.4-120)
Dose not available	6 (7)	1 (2)
Distant metastases	17 (18)	12 (26)
Chemotherapy*	48 (53)	6 (13)
Not reported	2 (2)	0 (0)
Doxorubicin weekly	10 (11)	1 (2)
Paclitaxel weekly	8 (9)	0 (0)
Cisplatin	3 (3)	0 (0)
Paclitaxel + carboplatin	22 (24)	5 (11)
Paclitaxel + pemetrexed	6 (7)	2 (4)
Doxorubicin based*	10 (11)	0 (0)
Other	4 (0)	0 (0)
unknown	2 (2)	0 (0)
More than one chemotherapeutic regimen	13 (14)	2 (4)
Tyrosine kinase inhibitor (TKI) and/or immune checkpoint inhibitor (ICI) therapy	15 (16)	13 (28)
Lenvatinib	3 (3)	9 (19)
Pembrolizumab	2 (2)	1 (2)
Lenvatinib + pembrolizumab	1 (1)	2 (4)
Other^#^	10 (11)	8 (17)
more than one therapy	1 (1)	5 (11)

*Chemotherapy other than monotherapy with doxorubicin weekly.

*Vemurafenib, Sunitinib, Cabozantinib, Pazopanib, Imatinib, Nivolumab.

ATC patient #1 (TPS: 10%, panFGFR expression: 2.8 mRNA/cell) patient received LEN (dose varying from 8 mg to 14 mg) after failure of RCT with partial remission (PR) as best overall response (BOR). LEN monotherapy was followed by LEN and PEM combination therapy (doses 4 mg and 200 mg respectively) with stable disease (SD) as BOR (PFS until after last-follow-up: 9.9 months).

ATC patient #2 (TPS: 10%) experienced SD by PEM monotherapy (dose: 200 mg every 3 weeks) with a PFS of 22.4 months. PEM treatment was followed by a combination of paclitaxel (80mg every week) and PEM for 3.0 months. The patient deceased one month after termination of treatment.

ATC patient #3 (TPS: 1%, panFGFR expression: 1.0 mRNA/cell) consecutively received LEN and PEM as single agents each with PD as BOR. The PFS for LEN and PEM treatment was 1.7 and 2.3 months, respectively.

In a fourth ATC patient (TPS: 95%, panFGFR expression: 3.1 mRNA/cell), treatment with LEN and PEM was started after failure of RCT five days before the patient deceased and hence is considered unevaluable.

ATC patient #5 (TPS: 70%, panFGFR expression: 1.0 mRNA/cell) received LEN (dose:24 mg) after failure of RCT with a PFS of 4.4 months with PD as BOR.

PDTC patient #6 (TPS: 50%, panFGFR expression not available) who was radioiodine refractory at diagnosis received LEN combined to PEM as first line treatment. Best response was SD and the patient received this combination for 21.5 months until progression occurred. Treatment was then switched to LEN and everolimus.

PDTC patient #7 (TPS: 30%, panFGFR expression 2.83 mRNA/cell) was treated with LEN, best response was SD and PFS 13.7 months. Everolimus was added to the therapy which resulted in SD for 9.7 months. Later, PEM was added to everolimus and LEN with PD after 1.8 months. The patient was still alive at last follow-up.

In a third (#8) PDTC patient (TPS: 90%, panFGFR expression not available) treatment was started with PEM as part of a clinical trial (Keynote 158) and discontinued 4 months later due to PD. Outside of the study, the patient received LEN and is still alive with SD as BOR after data cut-off (PFS until after last follow-up: 38.6 months).

PDTC patient #9 (TPS: 50%, panFGFR expression: 3.1 mRNA/cell) first received LEN (dose: 10 mg) with PR as BOR and PFS of 7.6 months followed by sorafenib (400 mg) for 3.0 months. Treatment was continued with LEN and PEM combination therapy with PR as BOR and a PFS of 6.6 months. Later the patient continued treatment with PEM monotherapy for 2.8 months.

PDTC patient #10 (TPS: 1%, panFGFR expression: 2.6 mRNA/cell) first received LEN (max. dose: 20 mg) with mixed response (MR) as BOR. Treatment was terminated due to adverse events with PFS of 5.7 months. LEN treatment was then followed by sorafenib (dose: 800 mg) for 1.7 months which was followed by chemotherapy.

Primary LEN monotherapy (max. dose: 14 mg) was also administered to PDTC patient #11 (TPS: 1%, panFGFR expression: 4.5 mRNA/cell) for 26.1 months. PEM was added shortly after imaging showed PD, but the patient deceased one month later.

**Figure 5:**
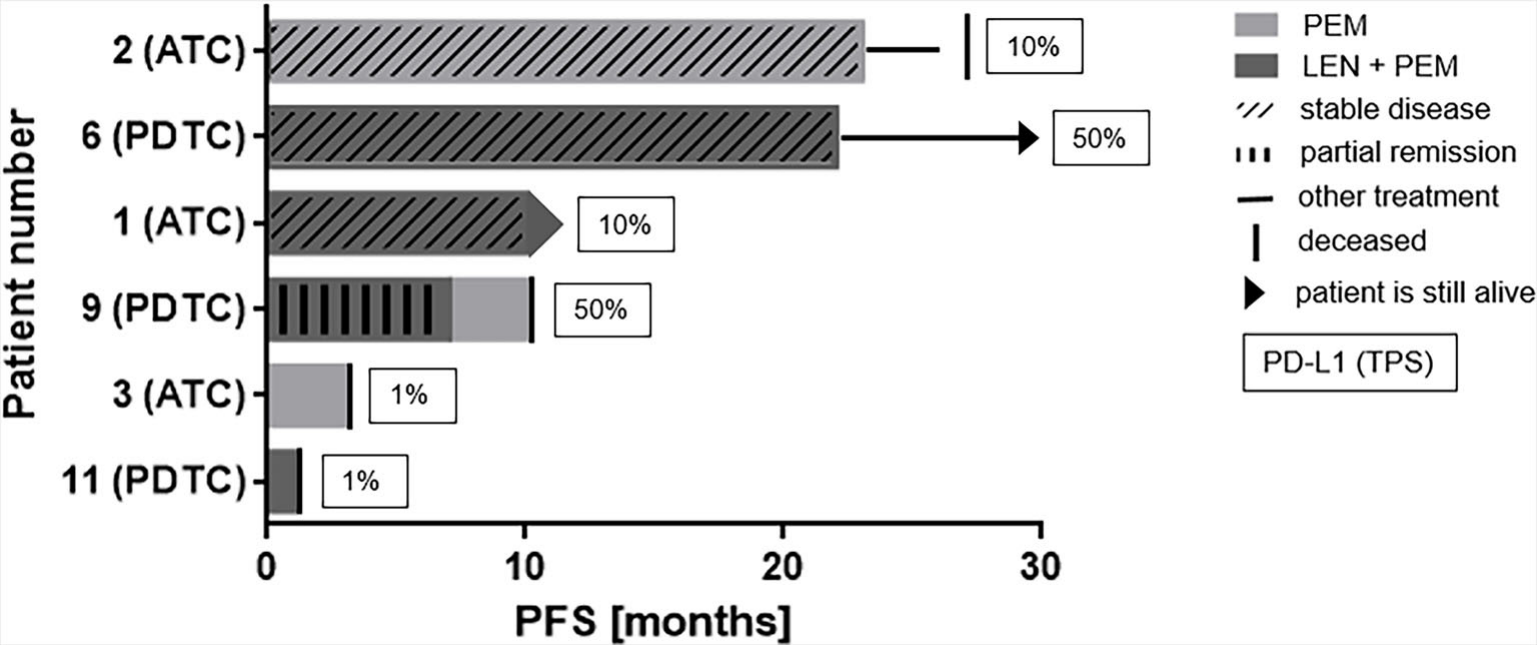
Swim lane plot demonstrating PFS on PEM or the combination of LEN and PEM: Figure shows data before and after data cut-off. Patients 6 and 1 are still alive with patient 1 still receiving LEN + PEM. Exact date of progress or exact treatment duration was not reported because PD was always followed by change of therapeutic regimen or death. Progressive disease was the best response in patients 9 and 3 while on PEM monotherapy and in patient 11 receiving LEN + PEM.

The PFS and treatment response of the different patients treated with PEM alone in combination with LEN is depicted in **Figure 5**. Patients with moderate or high expression showed longer PFS.

Four additional PDTC patients were treated with LEN monotherapy. One patient (TPS: 5%, panFGFR expression: 2.1 mRNA/cell) received LEN 24 mg (PFS: 10.5 months) with SD followed by cabozantinib. Another patient (TPS: 80%) received LEN (max. dose: 24 mg) after RCT treatment, but deceased one month after initiation. A third patient (TPS: 10%) was treated with LEN for 8.9 months and died 8 days later and a fourth patient (TPS: 10%) received LEN (max. dose: 24 mg) for 19.7 months at last follow-up after data cut-off and is still alive with SD.

### Prognostic Factors of Disease Specific Survival in ATC and PDTC

The association of clinical factors with DSS in ATC and PDTC is summarized in **Table 4**, respectively. In PDTC where prognostic factors are ill described, UICC stage I, II or III compared to IV, and use of RIT were associated with a significantly longer DSS. Treatment with external beam radiation therapy (EBRT) or chemotherapy was associated with a significantly longer DSS in ATC while the opposite was observed in PDTC, where the use of EBRT and chemotherapy most likely indicate aggressive clinical course.

**Table 4:** Impact of PD-L1 expression and clinical parameters on disease specific death from ATC.

Prognostic factors	Univariate analysis			Multivariate analysis		
	HR	95% CI	P (log rank)	HR	95% CI	P (cox regression)
**ATC**						
**Pretreatment factors**						
** Sex**						
Male (n=40)						
Female (n=53)			0.82			
** Age at diagnosis** (years)						
<69 (n=46)			** **			
≥69 (n=47)	1.689	1.091-2.614	**0.017**	1.724	0.958-3.104	0.069
** UICC**						
IVB (n=41)						
IVC (n=48)	1.886	1.200-2.966	**0.005**	2.068	1.244-3.438	**0.005**
** PD-L1**			0.495			
≤5% (n=25)						
5%-50% (n=29)						
≥50% (n=39)						
** Complete local resection**			0.392			
Yes (n=11)						
No (n=71)						
**Treatment factors**						
** External beam radiation**						
No (n=14)						
Yes (n=79)	0.503	0.277-0.915	**0.021**	0.463	0.228-0.941	**0.033**
** External beam radiation**						
<55 Gy (n=36)						
≥55 Gy (n=37)			0.117			
** Chemotherapy**						
No (n=43)		** **				
Yes (n=48)	0.619	0.399-0.962	**0.031**	0.582	0.335-1.011	0.055
**PDTC**						
**Pretreatment factors**						
** Sex**						
Male (n=17)						
Female (n=30)			0.338			
** Age at diagnosis** (years)						
<63 (n=23)						
≥63 (n=24)			0.48			
** UICC**						
I, II and III (n=33)						
IVA and IVC (n=13)	3.176	1.002-10.060	**0.04**	2.984	0.907-9.819	0.072
** PD-L1**			0.496			
≤5% (n=25)						
5%-50% (n=10)						
≥50% (n=12)						
** Complete local resection**						
Yes (n=12)			0.119			
No (n=25)						
**Treatment factors**						
** Radioiodine treatment**						
No (n=11)					0.07-2.177	
Yes (n=36)	0.148	0.038-0.517	**0.001**	0.284		0.284
** Radioiodine treatment**						
<6.65 GBq (n=18)						
≥6.65 GBq (n=18)			0.601			
** External beam radiation**						
No (n=31)		** **			0.512-12.702	
Yes (n=16)	4.065	1.180-14.001	**0.016**	2.55		0.253
** External beam radiation**						
<60.5 Gy (n=7)						
≥60.5 Gy (n=8)			0.531			
** Chemotherapy**						
No (n=41)					0.367-8.626	
Yes (n=6)	4.291	1.050-17.537	**0.027**	1.779		0.475

*p-values of Bonferroni testing for pairwise comparisons between ATC and PDTC are indicated.

**Figure 6:**
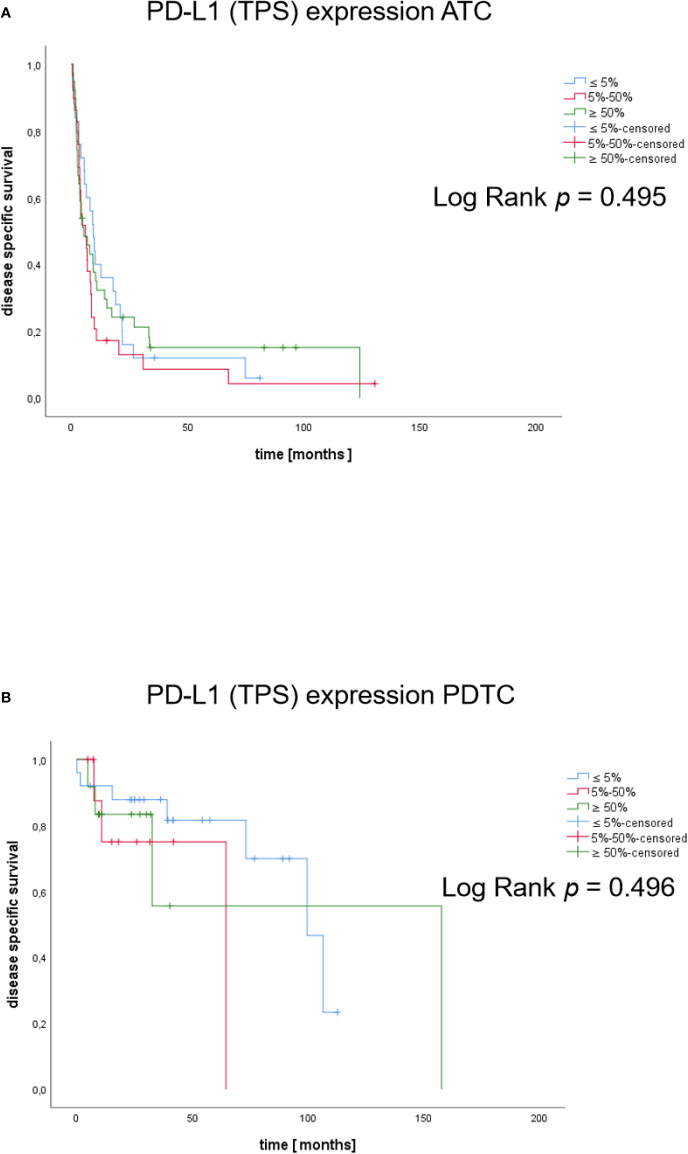
Kaplan-Meier plots of DSS in patients according to PD-L1 expression. Disease specific survival of ATC **(A)** and PDTC **(B)** patients with TPS categories ≤5%, 5-50%, ≥50%).

Considering PD-L1 TPS ≤ 5% as low, 6-49% intermediate and 50-100% as high expression, we did not observe significant differences of DSS among patients with ATC (p=0.495) or PDTC (p=0.496) patients (**Figure 6**). Using established cut-offs associated with response to PEM in NSCLC (scores from 0-49%: low; scores from 50-100%: high) no significant differences in DSS were observed as well (**Supplementary Figure 1**). We likewise did not find a prognostic relevance of FGFR1-4 expression for DSS (**Supplementary Figure 2**).

## Discussion

In this large multicenter analysis of prospectively and retrospectively collected clinical data and tissue specimens we systematically investigated the expression of the immune checkpoint molecule PD-L1 in the orphan diseases PDTC and ATC. We found variable expression in both types of aggressive thyroid malignancies with a significantly higher expression in ATC *vs.* PDTC. Importantly, while more than half of the PDTC samples showed a TPS below 5%, only 21% of ATC had PD-L1 below that arbitrary threshold. Of note, TPS of at least 1% was observed in all PDTC and ATC. In normal thyroid tissue, PD-L1 was absent but it was present at low levels in differentiated papillary thyroid carcinoma.

While clinical observations and small published studies suggested the expression of PD-L1 in the majority of ATC, we are not aware of a similarly large study on this topic (29, 30). Further strengths of our analysis are the systematic staining and evaluation at a single institution by a specialized endocrine pathologist and the systematic verification of diagnoses which is not the case in other series or was not reported (8). Overall, the presence of high PD-L1 expression in a substantial proportion of ATC and PDTC indicates a rationale for treatment with immune checkpoint inhibitors targeting the PD-1/PD-L1 axis. Our study is unable to support, however, a specific cut-off that would govern treatment with immune checkpoint inhibitors such as PEM. The TPS has been established as a response marker primarily for non-small cell lung cancer (NSCLC) and treatment of squamous cell head and neck cancer. In a retrospective analysis of ATC patients treated with kinase inhibitors and PEM progressive disease was observed in patients with PD-L1 TPS of 5%, 30% and 80% while a partial response was demonstrated in five patients, four of whom had PD-L1 TPS available with scores of **>**95%, 90%, 20% and **>**10% (8). Our data showed higher median PD-L1 expression in PTC than in PDTC (TPS 30% and 5% respectively), but range of PD-L1 expression in PDTC was much broader compared to PTC samples. PD-L1 expression has been reported for PTC tissue with and without lymphoid thyroiditis. PTC specimens with lymphoid thyroiditis showed higher expression of PD-L1 (39,1%) in comparison to PTC tissue without lymphoid thyroiditis (6,9%) (31). A review on the expression of PD-L1 in PTC tissue demonstrated significant association of PD-L1 expression with reduced disease-free survival (DFS), but not OS (32). We did not find any impact of PD-L1 expression on DSS. This is not surprising since the vast majority of patients did not receive PD-1/PD-L1 directed therapy. Hence, PD-L1 is not a prognostic marker but possibly a marker of treatment response in ATC and PDTC. Accordingly, a recently published ATC cohort from a phase I/II trial treated with spartalizumab, a PD-1 inhibitor, demonstrated objective response exclusively in patients with detectable PD-L1 expression (26). Guidelines recommend the evaluation of PD-L1 status in stage IVC ATC cases with lack of BRAF V600E mutation (6). Detection of high PD-L1 expression can be followed by treatment with immune checkpoint inhibitors and can be an alternative to chemotherapy and/or radiation (6).

The rationale to combine PD-L1 with LEN is supported by preclinical observations in an immunocompetent mouse model (33). The authors demonstrated profound changes of the immune microenvironment: LEN led to a pronounced increase in tumor-infiltrating immune cells, tumor-associated macrophages but also a pronounced increase of peripheral and tumoral polymorphonuclear myeloid derived suppressor cells (PMN-MDSC). The authors concluded that LEN exerts both pro-inflammatory and anti-inflammatory immune effects. By experimentally reducing the number of immunosuppressive PMN-MDSC the authors showed increased antitumoral efficacy of LEN alone and inhibition of the PD-1/PD-L1 axis was likewise associated with a decrease of immunosuppressive cell types. There is currently limited evidence of a direct immunomodulatory effect of LEN. Given the poor clinical response of ATC to VEGFR-directed TKI such as sorafenib, pazopanib and sunitinib, we reasoned that expression of the LEN targets FGFR1-4 on tumor cells may contribute to the specific antitumoral response of LEN monotherapy and in combination with immune checkpoint inhibitors. While immunohistochemistry of FGFR4 only has been used in one series (20) we consider our approach with RNAscope^®^ *in situ* hybridization more reliable because it is not susceptible to cross-reactivity of FGFR antibodies in immunohistochemistry and permits the quantification of individual FGFRs. We found extremely low expression of all FGFR in the samples studied. FGFR1 was expressed at highest levels in PDTC and ATC compared to normal thyroid and PTC samples. Overall, we found significantly higher expression of the combination of FGFR1-4 in PDTC and ATC compared to normal thyroid but not to PTC. Tumor infiltrating leukocytes did not express FGFR1-4. Although we cannot exclude that the low expression of FGFR1-4 is still biologically relevant, we conclude that tumoral FGFRs are unlikely to be involved in the immunostimulatory action of LEN.

Our study of PD-L1 and FGFR1-4 expression in ATC and PDTC has some limitations: First, our study is – in part - retrospective in nature. Thus, selection bias confounds the association of treatment factors with prognosis and is the cause of the shorter DSS in PDTC patients receiving chemotherapy or EBRT. On the other hand, radioiodine positive PDTC have an inherently better prognosis.

Second, only few patients were treated with LEN or PEM (12 and 3 respectively) and even fewer patients were primarily treated with a combined regimen (n=3). It is therefore impossible to define cut-off values of response. Third, the tumor mutational burden and the LEN targets c-kit, RET, VEGFR 1-3 or PDGFR-α were not analyzed because we considered FGFR as the most relevant drug-specific target molecules. Indeed it has been suggested that beyond the actual tumoral angiogenesis, patient factors may contribute to the antitumoral effects observed with multi-kinase inhibitors such as LEN but also sorafenib (34, 35).

Finally, although relatively large for the rarity of the disease, the number of PDTC patients is still limited and no specific selection of PDTCs refractory to RIT was applied.

Together with the available preclinical data, our findings suggest that the immunostimulatory effect of LEN in ATC and the promising finding of clinical activity associated with this drug may be conferred by direct effects on circulating immune cells. It is noteworthy that the combination of LEN and PEM has been approved for the treatment of endometrial cancer and is under study in a broad spectrum of tumors (36). Markers of treatment response are yet to be discovered.

## Conclusion

Our study of the expression of PD-L1 and FGFR 1-4 in ATC and PDTC supports the use of immune checkpoint inhibitors in the majority of ATC and some PDTC with high PD-L1 expression although the role of PD-L1 expression for treatment decisions remains to be established. Tumoral FGFR expression is similarly low in ATC and PDTC and likely not the principal target of lenvatinib. In the light of the clinical response of ATC to combined LEN and immune checkpoint inhibitor treatment, we suggest a yet unknown tumoral LEN target or a LEN target not expressed in the tumor to be relevant for the clinically observed drug synergism. We propose to study peripheral immune cells and tumor infiltrating leukocytes systematically in clinical trials to identify markers of treatment response and better understand the mechanistic basis of combination therapy.

## Data Availability Statement

The original contributions presented in the study are included in the article/**Supplementary Material**, further inquiries can be directed to the corresponding author.

## Ethics Statement

Written informed consent was obtained from the individual(s) for the publication of any potentially identifiable images or data included in this article.

## Author Contributions

MK, MF, CSp, CD, MS, and AZ designed the study. PA, SK, and IS performed experiments. SK, VK, EB, TK, BSa, WF, HB, CSm, AZ, BSi, SA, MS, CD, CSp, MF, and MK provided samples and data. PA and MK analyzed the data. PA, SK, VK, BSi, AZ, MF, and MK interpreted the data. PA and MK wrote a manuscript draft. All authors contributed to the article and approved the submitted version.

## Funding

This work was supported – in part - by the Else Kröner Fresenius Stiftung, grant number 2016-A96.

## Conflict of Interest

The authors declare that the research was conducted in the absence of any commercial or financial relationships that could be construed as a potential conflict of interest.

## Publisher’s Note

All claims expressed in this article are solely those of the authors and do not necessarily represent those of their affiliated organizations, or those of the publisher, the editors and the reviewers. Any product that may be evaluated in this article, or claim that may be made by its manufacturer, is not guaranteed or endorsed by the publisher.

## Supplementary Material

Supplementary Figure 1 Kaplan-Meier plots of DSS in patients according to PD-L1 expression. Disease specific survival of ATC **(A)** and PDTC **(B)** patients with TPS categories <50% and ≥50%).

Supplementary Figure 2 Kaplan-Meier plots of DSS in patients according to panFGFR expression. Disease specific survival of ATC **(A)** and PDTC **(B)** patients with mean panFGFR expression (2 mRNA/cell [ATC], 3 mRNA/cell [PDTC]).

## References

[1] Jemal A Bray F Center MM Ferlay J Ward E Forman D. Global Cancer Statistics. CA Cancer J Clin (2011) 61(2):69–90. 10.3322/caac.20107 21296855

[2] Sanders EM Jr. LiVolsi VA Brierley J Shin J Randolph GW. An Evidence-Based Review of Poorly Differentiated Thyroid Cancer. World J Surg (2007) 31(5):934–45. 10.1007/s00268-007-9033-3 17431717

[3] Owens PW McVeigh TP Fahey EJ Bell M Quill DS Kerin MJ. Differentiated Thyroid Cancer: How Do Current Practice Guidelines Affect Management? Eur Thyroid J (2018) 7(6):319–26. 10.1159/000493261 PMC6276740 30574463

[4] Wendler J Kroiss M Gast K Kreissl MC Allelein S Lichtenauer U. Clinical Presentation, Treatment and Outcome of Anaplastic Thyroid Carcinoma: Results of a Multicenter Study in Germany. Eur J Endocrinol (2016) 175(6):521–9. 10.1530/EJE-16-0574 27926471

[5] Maniakas A Dadu R Busaidy NL Wang JR Ferrarotto R Lu C. Evaluation of Overall Survival in Patients With Anaplastic Thyroid Carcinoma, 2000-2019. JAMA Oncol (2020) 6(9):1397–1404. 10.1001/jamaoncol.2020.3362 32761153 PMC7411939

[6] Bible KC Kebebew E Brierley J Brito JP Cabanillas ME Clark TJ Jr.. 2021 American Thyroid Association Guidelines for Management of Patients With Anaplastic Thyroid Cancer. Thyroid (2021) 31(3):337–86. 10.1089/thy.2020.0944 PMC8349723 33728999

[7] Smallridge RC Copland JA. Anaplastic Thyroid Carcinoma: Pathogenesis and Emerging Therapies. Clin Oncol (R Coll Radiol) (2010) 22(6):486–97. 10.1016/j.clon.2010.03.013 PMC3905320 20418080

[8] Iyer PC Dadu R Gule-Monroe M Busaidy NL Ferrarotto R Habra MA. Salvage Pembrolizumab Added to Kinase Inhibitor Therapy for the Treatment of Anaplastic Thyroid Carcinoma. J Immunother Cancer (2018) 6(1):68. 10.1186/s40425-018-0378-y 29996921 PMC6042271

[9] Ferrari SM Elia G Ragusa F Ruffilli I La Motta C Paparo SR. Novel Treatments for Anaplastic Thyroid Carcinoma. Gland Surg (2020) 9(Suppl 1):S28–s42. 10.21037/gs.2019.10.18 32055496 PMC6995904

[10] Subbiah V Kreitman RJ Wainberg ZA Cho JY Schellens JHM Soria JC. Dabrafenib and Trametinib Treatment in Patients With Locally Advanced or Metastatic BRAF V600-Mutant Anaplastic Thyroid Cancer. J Clin Oncol (2018) 36(1):7–13. 10.1200/JCO.2017.73.6785 29072975 PMC5791845

[11] Thiagarajan S Yousuf A Shetty R Dhar H Mathur Y Nair D. Poorly Differentiated Thyroid Carcinoma (PDTC) Characteristics and the Efficacy of Radioactive Iodine (RAI) Therapy as an Adjuvant Treatment in a Tertiary Cancer Care Center. Eur Arch Otorhinolaryngol (2020) 277(6):1807–14. 10.1007/s00405-020-05898-9 32170421

[12] Lee DY Won JK Lee SH Park DJ Jung KC Sung MW. Changes of Clinicopathologic Characteristics and Survival Outcomes of Anaplastic and Poorly Differentiated Thyroid Carcinoma. Thyroid (2016) 26(3):404–13. 10.1089/thy.2015.0316 26541309

[13] Tumours WHOCo. “WHO Classification of Tumours of Endocrine Organs”. In: Lloyd RV Osamura RY Klöppel G Rosai J, editors. World Health Organization Classification of Tumours, 4th ed. Lyon: International Agency for Research on Cancer IARC (2017). p. 100–6.

[14] Schlumberger M Tahara M Wirth LJ Robinson B Brose MS Elisei R. Lenvatinib Versus Placebo in Radioiodine-Refractory Thyroid Cancer. N Engl J Med (2015) 372(7):621–30. 10.1056/NEJMoa1406470 25671254

[15] Marotta V Sciammarella C Vitale M Colao A Faggiano A. The Evolving Field of Kinase Inhibitors in Thyroid Cancer. Crit Rev Oncol Hematol (2015) 93(1):60–73. 10.1016/j.critrevonc.2014.08.007 25240824

[16] Marotta V Chiofalo MG Di Gennaro F Daponte A Sandomenico F Vallone P. Kinase-Inhibitors for Iodine-Refractory Differentiated Thyroid Cancer: Still Far From a Structured Therapeutic Algorithm. Crit Rev Oncol Hematol (2021) 162:103353. 10.1016/j.critrevonc.2021.103353 34000414

[17] Iwasaki H Toda S Suganuma N Murayama D Nakayama H Masudo K. Lenvatinib vs. Palliative Therapy for Stage IVC Anaplastic Thyroid Cancer. Mol Clin Oncol (2020) 12(2):138–43. 10.3892/mco.2019.1964 PMC6951241 31929884

[18] Takahashi S Tahara M Ito K Tori M Kiyota N Yoshida K. Safety and Effectiveness of Lenvatinib in 594 Patients With Unresectable Thyroid Cancer in an All-Case Post-Marketing Observational Study in Japan. Adv Ther (2020) 37(9):3850–62. 10.1007/s12325-020-01433-8 PMC7444395 32676927

[19] Dai S Zhou Z Chen Z Xu G Chen Y. Fibroblast Growth Factor Receptors (FGFRs): Structures and Small Molecule Inhibitors. Cells (2019) 8(6):614. 10.3390/cells8060614 PMC6627960 31216761

[20] Yamazaki H Yokose T Hayashi H Iwasaki H Osanai S Suganuma N. Expression of Fibroblast Growth Factor Receptor 4 and Clinical Response to Lenvatinib in Patients With Anaplastic Thyroid Carcinoma: A Pilot Study. Eur J Clin Pharmacol (2020) 76(5):703–9. 10.1007/s00228-020-02842-y 32034430

[21] Stuhler V Maas JM Rausch S Stenzl A Bedke J. Immune Checkpoint Inhibition for the Treatment of Renal Cell Carcinoma. Expert Opin Biol Ther (2020) 20(1):83–94. 10.1080/14712598.2020.1677601 31587590

[22] Garon EB Rizvi NA Hui R Leighl N Balmanoukian AS Eder JP. Pembrolizumab for the Treatment of non-Small-Cell Lung Cancer. N Engl J Med (2015) 372(21):2018–28. 10.1056/NEJMoa1501824 25891174

[23] Ferris RL Licitra L. PD-1 Immunotherapy for Recurrent or Metastatic HNSCC. Lancet (2019) 394(10212):1882–4. 10.1016/S0140-6736(19)32539-5 31679948

[24] Finn RS Ryoo BY Merle P Kudo M Bouattour M Lim HY. Pembrolizumab As Second-Line Therapy in Patients With Advanced Hepatocellular Carcinoma in KEYNOTE-240: A Randomized, Double-Blind, Phase III Trial. J Clin Oncol (2019) Jco1901307:193–202. 10.1200/JCO.19.01307 31790344

[25] Yu Y Zeng D Ou Q Liu S Li A Chen Y. Association of Survival and Immune-Related Biomarkers With Immunotherapy in Patients With Non-Small Cell Lung Cancer: A Meta-Analysis and Individual Patient-Level Analysis. JAMA Netw Open (2019) 2(7):e196879. 10.1001/jamanetworkopen.2019.6879 31290993 PMC6625073

[26] Capdevila J Wirth LJ Ernst T Ponce Aix S Lin CC Ramlau R. PD-1 Blockade in Anaplastic Thyroid Carcinoma. J Clin Oncol (2020) 38(23):2620–7. 10.1200/JCO.19.02727 PMC7476256 32364844

[27] Chintakuntlawar AV Yin J Foote RL Kasperbauer JL Rivera M Asmus E. A Phase 2 Study of Pembrolizumab Combined With Chemoradiotherapy as Initial Treatment for Anaplastic Thyroid Cancer. Thyroid (2019) 29(11):1615–22. 10.1089/thy.2019.0086 31595822

[28] Dierks C Seufert J Aumann K Ruf J Klein C Kiefer S. The Lenvatinib/Pembrolizumab Combination is an Effective Treatment Option for Anaplastic and Poorly Differentiated Thyroid Carcinoma. Thyroid (2021) 31(7):1076–85. 10.1089/thy.2020.0322 PMC8290324 33509020

[29] Bastman JJ Serracino HS Zhu Y Koenig MR Mateescu V Sams SB. Tumor-Infiltrating T Cells and the PD-1 Checkpoint Pathway in Advanced Differentiated and Anaplastic Thyroid Cancer. J Clin Endocrinol Metab (2016) 101(7):2863–73. 10.1210/jc.2015-4227 PMC4929840 27045886

[30] Zwaenepoel K Jacobs J De Meulenaere A Silence K Smits E Siozopoulou V. CD70 and PD-L1 in Anaplastic Thyroid Cancer - Promising Targets for Immunotherapy. Histopathology (2017) 71(3):357–65. 10.1111/his.13230 28383817

[31] Fadia M Fookeerah P Ali S Shadbolt B Greenaway T Perampalam S. PD-L1 Expression in Papillary Thyroid Cancer With and Without Lymphocytic Thyroiditis: A Cross Sectional Study. Pathology (2020) 52(3):318–22. 10.1016/j.pathol.2019.11.007 32107082

[32] Girolami I Pantanowitz L Mete O Brunelli M Marletta S Colato C. Programmed Death-Ligand 1 (PD-L1) Is a Potential Biomarker of Disease-Free Survival in Papillary Thyroid Carcinoma: A Systematic Review and Meta-Analysis of PD-L1 Immunoexpression in Follicular Epithelial Derived Thyroid Carcinoma. Endocr Pathol (2020) 31(3):291–300. 10.1007/s12022-020-09630-5 32468210

[33] Gunda V Gigliotti B Ashry T Ndishabandi D McCarthy M Zhou Z. Anti-PD-1/PD-L1 Therapy Augments Lenvatinib’s Efficacy by Favorably Altering the Immune Microenvironment of Murine Anaplastic Thyroid Cancer. Int J Cancer (2019) 144(9):2266–78. 10.1002/ijc.32041 30515783

[34] Marotta V Sciammarella C Capasso M Testori A Pivonello C Chiofalo MG. Preliminary Data of VEGF-A and VEGFR-2 Polymorphisms as Predictive Factors of Radiological Response and Clinical Outcome in Iodine-Refractory Differentiated Thyroid Cancer Treated With Sorafenib. Endocrine (2017) 57(3):539–43. 10.1007/s12020-016-1200-6 27981515

[35] Marotta V Sciammarella C Capasso M Testori A Pivonello C Chiofalo MG. Germline Polymorphisms of the VEGF Pathway Predict Recurrence in Nonadvanced Differentiated Thyroid Cancer. J Clin Endocrinol Metab (2017) 102(2):661–71. 10.1210/jc.2016-2555 27849428

[36] Hao Z Wang P. Lenvatinib in Management of Solid Tumors. Oncologist (2020) 25(2):e302–e10. 10.1634/theoncologist.2019-0407 PMC7011622 32043789

[37] Geiger J Both S Kircher S Neumann M Rosenwald A Jahns R. Hospital-Integrated Biobanking as a Service – The Interdisciplinary Bank of Biomaterials and Data Wuerzburg (Ibdw). Open J Bioresour (2018) 5:6. 10.5334/ojb.38

