# Supplementary figures and images for: FGF-Receptors and PD-L1 in Anaplastic and Poorly Differentiated Thyroid Cancer: Evaluation of the Preclinical Rationale
Source: Front Endocrinol (Lausanne). 2021 Aug 12;12:712107. doi: 10.3389/fendo.2021.712107 (PMC8406771; doi:10.3389/fendo.2021.712107)

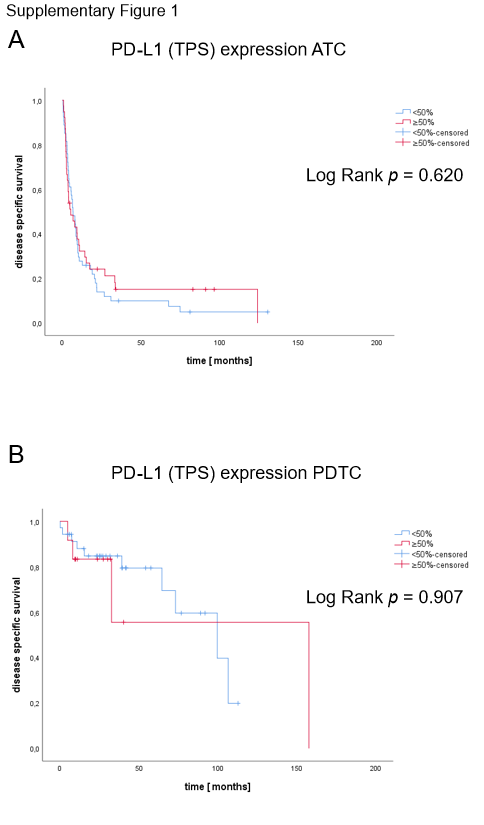

Supplement: Supplementary Figure 1 — Kaplan-Meier plots of DSS in patients according to PD-L1 expression. Disease specific survival of ATC (A) and PDTC (B) patients with TPS categories <50% and ≥50%). [file Image_1.tiff]

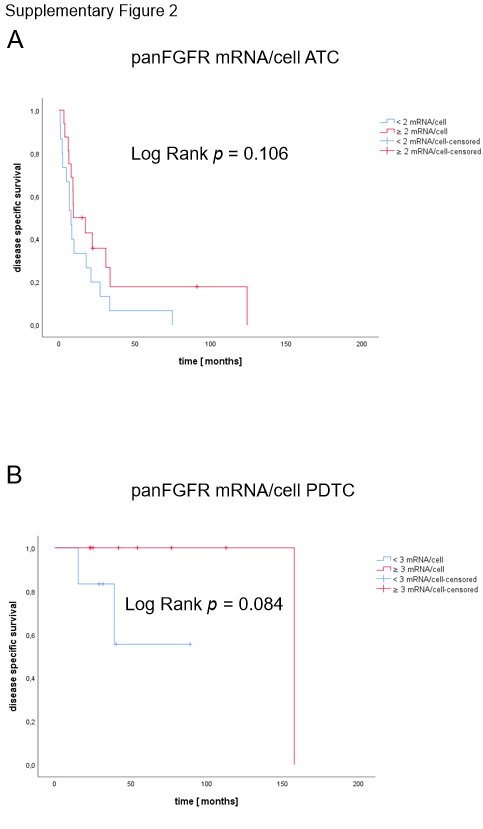

Supplement: Supplementary Figure 2 — Kaplan-Meier plots of DSS in patients according to panFGFR expression. Disease specific survival of ATC (A) and PDTC (B) patients with mean panFGFR expression (2 mRNA/cell [ATC], 3 mRNA/cell [PDTC]). [file Image_2.tiff]
